# Mutism. What to expect?

**DOI:** 10.1192/j.eurpsy.2022.1507

**Published:** 2022-09-01

**Authors:** I. Santos Carrasco, J. Gonçalves Cerejeira, M. Fernández Lozano, A. Gonzaga Ramírez, M. Queipo De Llano De La Viuda, G. Guerra Valera, C. Vallecillo Adame, C. De Andrés Lobo, T. Jiménez Aparicio, B. Rodríguez Rodríguez, N. Navarro Barriga, M.J. Mateos Sexmero, E. Pérez, L. Gallardo Borge

**Affiliations:** 1 Clinical Hospital of Valladolid, Psychiatry, Valladolid, Spain; 2 Hospital Clínico Universitario de Valladolid, Psychiatry, Valladolid, Spain; 3 Hospital Clínico Universitario de Valladolid, Psiquiatría, VALLADOLID, Spain; 4 Hospital Clínico Universitario, Psiquiatría, Valladolid, Spain

**Keywords:** Catatonia, mutism, emergency room

## Abstract

**Introduction:**

Mutism is the inability or unwillingness to speak, resulting in an absence or marked paucity of verbal output. Mutism is a common manifestation of psychiatric, neurological, and drug-related illnesses. Psychiatric disorders associated with mutism include schizophrenia, affective disorders, conversion reactions, dissociative states, and dementias. Neurological disorders causing mutism affect the basal ganglia, frontal lobes, or the limbic system.

**Objectives:**

Outline the importance of setting a differential diagnosis of mutism in the Emergency Room.

**Methods:**

Review of scientific literature based on a relevant clinical case.

**Results:**

Male, 58 years old. He has lived in a residence for 3 months due to voluntary refusal to ingest. Diagnosed with paranoid personality disorder. He is refered to the Emergency Service due to sudden mutism. During this day, he has been stable and suitable with a good functionality. For 3 hours he is mutist, oppositional attitude and stiff limbs, refusing to obey simple orders. Hyperalert and hyperproxia. Not staring. After ruling out organic pathology: normal blood tests, negative urine toxins and cranial CT without alterations, he was admitted to Psychiatry for observation and, finally, he was diagnosed with Psychotic Disorder NOS.

**Conclusions:**

Mutism most often occurs in association with other disturbances in behavior, thought processes, affect, or level of consciousness. The most common disorder of behavior occurring with mutism is catatonia. The differential diagnosis of mutism is complex. In some cases the diagnosis will be clarified only by careful observation and after a neurological evaluation. Published studies show neurological disorders presenting with mutism can be misdiagnosed as psychiatric.

**Disclosure:**

No significant relationships.

